# Evaluation of Serum Prolidase as a Biomarker and the Effects of Nintedanib in an Experimental Rat Model of Silicosis

**DOI:** 10.3390/jcm15145621

**Published:** 2026-07-17

**Authors:** Ezgi Nur Aydın, Dilek Ergün, Recai Ergün, Bahadır Öztürk, Mehmet Burak Ateş, Selman Turgut Koçak, Menendi Merve Savaş Güvendik

**Affiliations:** 1 Department of Pulmonary Medicine, Faculty of Medicine, Selçuk University, Konya 42130, Turkey; dilekkirbiyik@gmail.com (D.E.); recaiergun@gmail.com (R.E.); 2 Department of Biochemistry, Faculty of Medicine, Selçuk University, Konya 42130, Turkey; bahadirozturk@selcuk.edu.tr (B.Ö.); selturkoc@gmail.com (S.T.K.); 3 Department of Pathology, Faculty of Veterinary Medicine, Selçuk University, Konya 42130, Turkey; mehmetburakates@selcuk.edu.tr (M.B.A.); menendimerve123@gmail.com (M.M.S.G.)

**Keywords:** silicosis, prolidase, nintedanib, pulmonary fibrosis, biomarkers

## Abstract

**Background/Objectives**: Silicosis is a progressive occupational lung disease characterized by persistent inflammation and irreversible pulmonary fibrosis. Reliable circulating biomarkers for the early detection of fibrotic remodeling and monitoring of disease progression remain limited. Prolidase, a key enzyme involved in collagen turnover and extracellular matrix remodeling, has emerged as a potential biomarker in fibrotic disorders. Therefore, this study aimed to evaluate the potential role of serum prolidase as a biomarker for fibrotic remodeling and treatment monitoring in an experimental rat model of silicosis. **Methods**: Male Wistar rats were divided into three groups: control, silicosis, and silicosis plus nintedanib. Silicosis was induced by intratracheal crystalline silica instillation. Nintedanib was administered by gastric gavage at 50 mg/kg/day from day 10 to day 30. Lung tissues were evaluated histopathologically using hematoxylin and eosin and Masson’s trichrome staining. Serum prolidase, fibroblast growth factor-2 (FGF-2), platelet-derived growth factor (PDGF), and transforming growth factor-beta (TGF-β) levels were measured by enzyme-linked immunosorbent assay (ELISA). **Results**: Histopathological injury scores were significantly increased in the silicosis group and were attenuated by nintedanib treatment. Serum prolidase levels were significantly higher in both silica-exposed groups than in controls (*p* = 0.017), but did not differ significantly between untreated and nintedanib-treated animals. TGF-β, FGF-2, and PDGF levels were also significantly elevated following silica exposure. Serum prolidase showed a strong positive correlation with TGF-β (r = 0.69, *p* < 0.001). ROC analysis demonstrated good diagnostic performance for prolidase (AUC = 0.877), while the combined Prolidase + TGF-β model further improved discrimination (AUC = 0.969). The internally validated multivariable biomarker panel maintained high diagnostic accuracy after leave-one-out cross-validation (LOOCV) (AUC = 0.892). **Conclusions**: Serum prolidase may serve as a potential circulating biomarker of silica-associated fibrotic remodeling. Although nintedanib improved histopathological lung injury, persistently elevated prolidase levels suggest ongoing extracellular matrix remodeling rather than short-term treatment response. Further longitudinal experimental and clinical studies are warranted to validate its diagnostic and prognostic utility.

## 1. Introduction

Silicosis is a progressive occupational lung disease caused by the inhalation of respirable crystalline silica particles [1]. Despite being a preventable condition, it remains an important global health problem, particularly among workers in mining, construction, stone cutting, sandblasting, ceramic production, and similar high-risk industries [2]. The disease is characterized by persistent inflammation, oxidative stress, fibroblast activation, and excessive extracellular matrix deposition, ultimately leading to irreversible pulmonary fibrosis [3]. Depending on the intensity and duration of exposure, silicosis may present in acute, accelerated, or chronic forms, and disease progression can continue even after cessation of silica exposure [4]. In advanced stages, patients may develop worsening dyspnea, impaired pulmonary function, respiratory failure, and increased susceptibility to infections such as tuberculosis [5].

The clinical management of silicosis is challenging because there is currently no definitive curative treatment capable of reversing established pulmonary fibrosis [6]. Therefore, early recognition of disease activity, accurate assessment of progression, and objective monitoring during follow-up are crucial [7]. Conventional evaluation mainly relies on occupational history, radiological findings, pulmonary function tests, symptom assessment, and clinical follow-up [8]. However, these parameters may not fully reflect ongoing molecular and fibrotic activity, especially in the early or subclinical stages of disease progression. This limitation has increased interest in circulating biomarkers that may provide additional information about inflammation, oxidative stress, tissue remodeling, and fibrotic burden in patients with silicosis [9].

Prolidase is a cytosolic exopeptidase that catalyzes the final step of collagen degradation by hydrolyzing imidodipeptides containing C-terminal proline or hydroxyproline. Through its intracellular enzymatic activity, prolidase contributes to proline recycling, collagen turnover, extracellular matrix remodeling, inflammation, and tissue repair. Because collagen metabolism is fundamental to fibrotic processes, intracellular prolidase plays an essential role in maintaining extracellular matrix homeostasis [10,11].

In contrast, circulating (serum) prolidase levels are considered an indirect indicator of collagen metabolism and extracellular matrix remodeling rather than a direct reflection of intracellular enzyme activity [10]. Altered serum prolidase activity has been associated with oxidative stress, inflammation, and several fibrosis-related disorders, suggesting that it may serve as a non-invasive biomarker of tissue remodeling [10,12]. Therefore, serum prolidase has attracted increasing interest as a potential biomarker in fibrotic diseases, although its role in silicosis remains insufficiently investigated [13].

Although the pathogenesis of silicosis involves persistent inflammation, oxidative injury, fibroblast activation, and progressive collagen deposition, the potential utility of prolidase in silicosis has not been sufficiently clarified. In particular, its relationship with silica-induced fibrotic remodeling, treatment-related histopathological changes, and profibrotic biomarkers requires further investigation. Nintedanib is an intracellular tyrosine kinase inhibitor that targets multiple profibrotic signaling pathways, including platelet-derived growth factor (PDGF), fibroblast growth factor (FGF), and vascular endothelial growth factor (VEGF) receptors. It has been approved for the treatment of idiopathic pulmonary fibrosis and other progressive fibrosing interstitial lung diseases, where it slows the decline in lung function [14,15]. Although nintedanib is not currently approved for the treatment of silicosis, experimental studies have demonstrated that it attenuates silica-induced pulmonary fibrosis by reducing fibroblast activation, extracellular matrix deposition, and collagen accumulation, supporting its potential therapeutic role in silicosis [16].

The aim of the present study was to evaluate serum prolidase levels in an experimental rat model of silicosis and to investigate its potential role as a biomarker for diagnosis and treatment monitoring. We also aimed to examine the association of prolidase with histopathological injury, collagen deposition, and fibrosis-associated proteins, including TGF-β, FGF-2, and PDGF, thereby assessing its possible value as a circulating biomarker in silica-induced pulmonary fibrosis.

## 2. Materials and Methods

### 2.1. Ethical Approval

This experimental study was approved by the Local Ethics Committee for Animal Experiments of Selçuk University, Türkiye (approval no: 2025-12). All experimental procedures were conducted in accordance with national regulations and institutional guidelines for animal welfare. Appropriate anesthesia and postoperative monitoring were applied throughout the study to minimize pain, distress, and suffering.

### 2.2. Study Design and Experimental Groups

The study was designed as an experimental animal model of silicosis consisting of three groups: control, silicosis, and silicosis plus nintedanib. The control group received only intratracheal phosphate-buffered saline (PBS) and a vehicle. The silicosis group received only intratracheal crystalline silica and a vehicle. The silicosis plus nintedanib group received intratracheal crystalline silica, and nintedanib treatment was initiated on day 10 following silica instillation. Initially, 15 rats were assigned to each experimental group. Following the intratracheal silica instillation, 11 rats died on the day of the procedure due to procedure-related complications, and 3 more rats died within the first week. In accordance with the approved animal ethics protocol, untreated animals initially assigned to the control group—which had not yet undergone any intervention—were subsequently reassigned to the silica-exposed groups to preserve the planned experimental design. As a result, the study was completed with 5 rats in the control group, 13 rats in the silicosis group, and 13 rats in the silicosis plus nintedanib group.

Early deaths occurred before randomization to the experimental groups and before the initiation of nintedanib treatment. All surviving animals were monitored following intratracheal silica instillation, and randomization to the silica-only group and the silica plus nintedanib group was performed only after the experimental model was successfully established. Complete necropsies were performed on all animals that died prematurely. Histopathological examination of the lungs revealed only acute pulmonary congestion (hyperemia) and edema, without evidence of established silicotic nodules, granulomatous inflammation, or pulmonary fibrosis, consistent with deaths occurring during the immediate post-instillation period rather than as a consequence of silica-induced fibrotic lung disease.

### 2.3. Animals and Housing Conditions

Male Wistar rats weighing 200 ± 20 g and aged 8–10 weeks were used in the study. The animals were obtained from Selçuk University Experimental Medicine Research and Application Center, Konya, Türkiye. Rats were housed in sterile cages under controlled environmental conditions, including a temperature of 24–26 °C, relative humidity of 60–70%, and a 12-h light/dark cycle. Standard pellet chow and water were provided ad libitum. Cage conditions were maintained in accordance with specific pathogen-free standards.

### 2.4. Preparation of Crystalline Silica

Crystalline silica CAS 7631-86-9; mean particle diameter: 1.6 μm; purity: 99%; Merck, Darmstadt, Germany; Cat. No. 107739 was used to establish the experimental silicosis model. To remove potential endotoxin contamination, crystalline silica was heated at 180–200 °C for at least 2 h. The silica particles were then suspended in sterile phosphate-buffered saline. Before administration, the suspension was sonicated for at least 10–15 min to ensure homogeneity and vortexed immediately before use.

### 2.5. Establishment of the Experimental Silicosis Model

The experimental silicosis model was established by single-dose intratracheal instillation of crystalline silica suspension, based on previously described silica-induced pulmonary fibrosis models [17]. This model mimics respiratory exposure to silica particles and is widely used to induce pulmonary inflammation, granulomatous reaction, and fibrotic changes in lung tissue. Before the procedure, rats were prepared after a short fasting period. Anesthesia was induced with 2% sodium pentobarbital administered intraperitoneally. After confirming an adequate depth of anesthesia, the animals were fixed in the supine position. The cervical region was disinfected with an antiseptic solution, and the procedure was performed under sterile conditions. A small cervical incision was made, and the trachea was carefully exposed. Crystalline silica was suspended in sterile PBS at a concentration of 600 mg/mL. The suspension was mixed thoroughly by vortexing immediately before administration to ensure a homogeneous distribution of particles. A 20 μL volume of the prepared silica suspension was slowly and carefully instilled directly into the tracheal lumen using a fine-tipped micropipette or an appropriately calibrated syringe. Particular attention was paid to prevent aspiration, leakage, or reflux during instillation. In the control group, the same surgical procedure was performed; however, an equal volume of sterile PBS was administered intratracheally instead of silica suspension. This approach allowed control of the potential effects of the surgical procedure and intratracheal instillation itself. After completion of the instillation, the cervical incision was closed under sterile conditions. The animals were kept in a heated environment and monitored until full recovery from anesthesia. Respiratory pattern, general behavior, and clinical condition were observed at regular intervals after the procedure.

### 2.6. Nintedanib Treatment

Rats in the silicosis plus nintedanib group received nintedanib esylate (OFEV^®^ 100 mg capsule Boehringer Ingelheim, Ingelheim am Rhein, Germany; barcode no: 8699693190079) at a dose of 50 mg/kg/day by gastric gavage. The selected dose was based on previous experimental evidence demonstrating the antifibrotic efficacy of nintedanib in pulmonary fibrosis models and its inhibitory effects on profibrotic signaling pathways, including PDGF, FGF, and VEGF receptor-mediated pathways [14]. Treatment was initiated on day 10 after silica instillation and continued once daily until day 30, for a total duration of 21 days. During the same period, rats in the control and silicosis groups received an equal volume of vehicle solution.

### 2.7. Blood and Tissue Sampling

Thirty days after intratracheal silica instillation, corresponding to the termination day of the experimental protocol, blood samples were collected from the animals under anesthesia. Blood samples were allowed to clot and then centrifuged to separate serum. Serum samples were stored at −80 °C until biochemical analyses. Following euthanasia, lung tissues were excised and fixed in 10% neutral-buffered formalin for histopathological examination.

### 2.8. Histopathological Examination

After fixation, lung tissue samples were processed routinely and embedded in paraffin blocks. Sections with a thickness of 5 μm were obtained from paraffin-embedded tissues. Hematoxylin and eosin staining was performed to evaluate inflammatory changes, whereas Masson’s trichrome staining was used to assess collagen deposition and pulmonary fibrosis.

### 2.9. Histopathological Scoring

Histopathological evaluation was performed in a blinded manner, independent of group allocation. The following parameters were evaluated semi-quantitatively in each animal: inflammation 0–4, mast cell/eosinophil density 0–3, silicotic granulomatous nodule formation 0–4, alveolar architectural distortion 0–3, and fibrosis 0–4. A total lesion score was calculated by summing the scores of these parameters, resulting in a total score ranging from 0 to 18. The total lesion score was considered the primary histopathological outcome.

### 2.10. Biomarker Measurements

Serum levels of prolidase, platelet-derived growth factor (PDGF), fibroblast growth factor-2 (FGF-2), and transforming growth factor-beta (TGF-β) were measured using rat-specific commercial enzyme-linked immunosorbent assay ELISA kits according to the manufacturer’s instructions. The kit codes were as follows: prolidase E1224Ra, PDGF E0691Ra, FGF-2 E0852Ra, and TGF-β E0778Ra; BTLAB, China. Measurements were performed using a microplate reader at the appropriate wavelength, and concentrations were calculated based on standard curves. All ELISA measurements were performed in duplicate, and the mean values were used for statistical analysis.

### 2.11. Statistical Analysis

Statistical analyses were performed using IBM SPSS Statistics for Windows, version 27.0 (IBM Corp., Armonk, NY, USA). The distribution of continuous variables was assessed using the Shapiro–Wilk test. Descriptive statistics were presented as mean±standard error for normally distributed variables and as median (25th–75th percentile) for non-normally distributed variables. Histopathological scores were compared among the three experimental groups using one-way analysis of variance (ANOVA), followed by Tukey’s honestly significant difference (HSD) post hoc test for pairwise comparisons. Serum biomarker levels, including prolidase, FGF-2, PDGF, and TGF-β, were compared among groups using the Kruskal–Wallis test. When a significant difference was detected, pairwise comparisons were performed using the Mann–Whitney U test with Bonferroni correction. The relationships between serum prolidase levels and fibrosis-associated proteins were evaluated using Spearman’s rho correlation analysis. ROC analysis was conducted to evaluate the diagnostic performance of biomarkers between the silicosis group and the control group. In the analyses, the biomarkers prolidase, TGF-β, FGF-2, and PDGF were evaluated separately; for each, the area under the curve (AUC), optimal cutoff value, sensitivity, and specificity were calculated. Optimal cut-off values were determined based on the “closest-to-top-left” criterion on the ROC curve. In addition, Prolidase and TGF-β were used to construct a multivariate biomarker panel using logistic regression analysis in a combined model. The performance of the panel model was evaluated based on both the apparent model results and the internal validation results obtained using the Leave-One-Out Cross-Validation (LOOCV) method.

## 3. Results

### 3.1. Histopathological Findings

Histopathological scores differed significantly among the experimental groups. The silicosis group showed significantly higher inflammation, mast cell/eosinophil density, silicotic granulomatous nodule formation, alveolar architectural distortion, fibrosis, and total lesion scores compared with the control group. Nintedanib treatment significantly attenuated silica-induced histopathological injury, as reflected by lower inflammation, nodule formation, fibrosis, and total lesion scores compared with the untreated silicosis group. However, silicotic granulomatous nodule formation and total lesion score remained higher in the silicosis plus nintedanib group than in controls, indicating partial but not complete histopathological recovery (Table 1, Figure 1, Figure 2 and Figure 3A,B).

### 3.2. Serum Biomarker Levels

Serum biomarker levels differed significantly among the experimental groups. FGF-2, PDGF, prolidase, and TGF-β levels were significantly higher in the silica-exposed groups than in the control group. Prolidase levels were increased in both the silicosis and silicosis plus nintedanib groups compared with controls; however, no significant difference was observed between the untreated silicosis and nintedanib-treated groups (Table 2, Figure 3C–F)).

Correlation analysis showed a strong positive correlation between serum prolidase and TGF-β levels. In contrast, prolidase levels were not significantly correlated with FGF-2 or PDGF levels (Table 3, Figure 3G).

Upon examination of Spearman’s rho correlation heat map, it is observed that FGF-2 has higher correlation coefficients with all histopathological parameters compared to other biomarkers. In comparison, Prolidase, TGF-β, and PDGF were found to show positive correlations with histopathological findings ranging from low to moderate (Figure 3H).

The increase in histopathological damage parallels changes in serum biomarker levels. In particular, FGF-2 stands out as the biomarker that most strongly reflects histopathological damage, exhibiting the highest correlation coefficients with histopathological parameters. In addition, the strong positive correlation identified between Prolidase and TGF-β supports the notion that these two biomarkers change in tandem.

FGF-2 demonstrated the highest diagnostic performance in distinguishing between the silicosis group and the control group (AUC = 1.000). TGF-β (AUC = 0.892), Prolidase (AUC = 0.877), and PDGF (AUC = 0.831) also exhibited high discriminatory performance. The discriminatory power of the combined model, which uses both prolidase and TGF-β, increased, reaching an AUC of 0.969. The multivariate biomarker panel model developed using logistic regression demonstrated excellent classification performance (AUC = 1.000); following internal validation via LOOCV, it maintained high diagnostic accuracy, achieving an AUC of 0.892, 92% sensitivity, and 100% specificity (Table 4, Figure 4).

## 4. Discussion

In the present study, intratracheal crystalline silica administration successfully induced a prominent experimental silicosis phenotype characterized by increased inflammation, silicotic granulomatous nodule formation, alveolar architectural distortion, fibrosis, and total lesion score. These findings are consistent with the study by Jiao et al., who established a rat silicosis model using intratracheal silica instillation and evaluated lung tissue after 45 days. In their study, Wistar rats were divided into silica-exposed and control groups, and the authors demonstrated increased fibrosis together with elevated pulmonary expression of CTGF, TGF-β1, and PDGF by immunohistochemistry and RT-PCR. They also reported positive correlations among CTGF, TGF-β1, and PDGF, supporting the central role of profibrotic growth factor signaling in silica-induced lung injury [18]. Similarly, Wei et al. investigated a silica-induced rat pulmonary fibrosis model and showed that bone marrow-derived mesenchymal stem cells attenuated lung injury by inhibiting inflammation and epithelial–mesenchymal transition. Their findings emphasized that silica exposure produces a combined inflammatory and fibrotic response rather than a purely structural lesion [19]. In line with these studies, our histopathological data confirm that the experimental model reproduced the key pathological features of silicosis, including granulomatous inflammation, collagen deposition, and architectural distortion. The significant increase in TGF-β, PDGF, and FGF-2 in our silica-exposed groups further supports the biological consistency between our model and previously reported silica-induced pulmonary fibrosis models.

The partial histopathological improvement observed in the nintedanib-treated group is also supported by previous antifibrotic studies in experimental silicosis. Da Silva et al. developed an inhaled nintedanib nanosuspension for experimental silicosis and established the model by single intratracheal instillation of silica microparticles at 800 mg/kg in C57BL/6 mice. Their study showed that localized nintedanib delivery improved several pathological features of silicosis without notable local or systemic toxicity, suggesting that tyrosine kinase inhibition may have therapeutic relevance in silica-induced fibrosis [16]. In another mechanistically related study, Shen et al. demonstrated that apelin prevented and alleviated crystalline silica-induced pulmonary fibrosis by inhibiting TGF-β1-triggered fibroblast activation [20]. They reported that apelin levels were decreased in silicosis patients and silica-induced fibrotic mice, and that both preventive treatment from day 3 and therapeutic treatment from day 15 attenuated silica-induced pulmonary fibrosis in mice. Our results are compatible with these observations because nintedanib significantly reduced histopathological injury and collagen deposition, although it did not normalize all parameters completely. Importantly, serum prolidase remained elevated in the nintedanib-treated group, suggesting that despite histological improvement, active matrix remodeling may persist during treatment. This finding indicates that prolidase may reflect ongoing fibrotic turnover rather than complete reversal of established silica-induced injury.

Several recent experimental studies have shown that suppression of inflammation and profibrotic signaling can attenuate silica-induced lung damage. Zhang Y. et al. investigated baicalin in a rat model of silica-induced lung inflammation and fibrosis and reported that baicalin alleviated pathological injury by inhibiting the TLR4/NF-κB pathway [21]. This is relevant to our findings because the silica group showed marked inflammatory infiltration and increased histopathological injury scores, whereas the nintedanib-treated group showed lower inflammatory and fibrotic scores. Ma et al. evaluated tetrandrine in silica-induced pulmonary fibrosis using a network pharmacology approach combined with experimental validation and reported that tetrandrine alleviated silica-induced fibrosis through the PI3K/AKT pathway [22]. Although baicalin, tetrandrine, and nintedanib act through different molecular pathways, all three models converge on the same pathological endpoint: attenuation of inflammation, fibroblast activation, and extracellular matrix accumulation. Our study adds to this evidence by showing that nintedanib reduced inflammation, fibrosis, and total lesion score in an experimental silicosis model. However, the persistence of higher prolidase and profibrotic biomarker levels in the treated group suggests that biochemical remodeling activity may continue even when histological injury is partially reduced. The elevation of serum prolidase in both silica-exposed groups is biologically plausible because prolidase is closely related to collagen degradation, proline recycling, and extracellular matrix turnover.

Previous studies have demonstrated that serum prolidase activity is altered in several fibrotic disorders, including liver fibrosis, systemic sclerosis, idiopathic pulmonary fibrosis, and cardiac fibrosis. Because prolidase plays a central role in collagen turnover and extracellular matrix remodeling, increased serum prolidase activity has generally been associated with active fibrogenesis and tissue remodeling. However, the direction and magnitude of these changes may vary according to the underlying disease, stage of fibrosis, and inflammatory activity. Therefore, prolidase should be interpreted as a marker of extracellular matrix remodeling rather than a disease-specific biomarker [23,24,25,26]. Our findings further support the potential role of prolidase as an indicator of fibrotic activity in silicosis.

Türkbeyler et al. specifically evaluated prolidase activity in an experimental lung fibrosis model. In their study, 30 male Wistar rats were randomly divided into three groups, with 10 rats in each group: control, bleomycin-induced lung fibrosis, and bleomycin-induced lung fibrosis treated with palosuran, a urotensin-II receptor antagonist [27]. They reported increased prolidase activity during the development of lung fibrosis and suggested that prolidase may serve as a marker for diagnosis and treatment monitoring in fibrotic lung disease. In parallel, Zhang F. et al. recently showed that trigonelline hydrochloride attenuated silica-induced pulmonary fibrosis by modulating fibroblast-to-myofibroblast differentiation, a key step in extracellular matrix accumulation and fibrotic remodeling [28]. These two studies support the interpretation that prolidase is not merely a nonspecific serum change but may reflect active collagen turnover and fibroblast-driven matrix remodeling. In our study, prolidase levels were significantly higher in both the silicosis and silicosis plus nintedanib groups than in controls, and prolidase showed a strong positive correlation with TGF-β (Spearman’s rho = 0.69, *p* < 0.001). This relationship strengthens the hypothesis that prolidase is closely associated with the profibrotic remodeling axis in silicosis. Nevertheless, because prolidase did not differ significantly between untreated and nintedanib-treated silicosis groups, its role may be stronger as a marker of silica-associated fibrotic remodeling than as a short-term marker of treatment response.

Although the present study was not designed to investigate the molecular mechanisms underlying prolidase regulation, several pathways may explain the observed increase in serum prolidase levels during silica-induced fibrosis. Silica exposure induces persistent oxidative stress, activation of alveolar macrophages, and increased production of profibrotic cytokines, particularly transforming growth factor-β (TGF-β) [29]. TGF-β stimulates fibroblast activation, myofibroblast differentiation, and excessive extracellular matrix deposition, processes that are closely associated with increased collagen turnover. Because prolidase is a key enzyme involved in collagen degradation and proline recycling, increased prolidase activity may reflect accelerated extracellular matrix remodeling during fibrogenesis rather than acting as a direct mediator of fibrosis. Further mechanistic studies investigating TGF-β/Smad, PI3K/Akt, and other profibrotic signaling pathways are warranted to better define the biological role of prolidase in silicosis [22,30].

The biomarker profile observed in our study is consistent with the growing interest in circulating markers for silicosis diagnosis and monitoring. Nong et al. conducted a case–control study evaluating serum Krebs von den Lungen-6 (KL-6) as a potential biomarker for early silicosis diagnosis and concluded that KL-6 may have diagnostic utility, particularly when combined with other clinical or laboratory parameters [31]. Similarly, Lombardi et al. evaluated inflammatory markers in individuals exposed to silica and assessed their association with silicosis and lung function impairment, using occupational history, clinical evaluation, pulmonary function testing, imaging findings, and circulating inflammatory markers [32]. These studies highlight the clinical need for noninvasive biomarkers that can complement imaging and functional assessment in silicosis. In the present study, serum prolidase demonstrated good diagnostic performance for distinguishing experimental silicosis from healthy controls (AUC = 0.877). TGF-β and FGF-2 also showed high diagnostic accuracy, with FGF-2 achieving perfect discrimination (AUC = 1.000). Furthermore, combining prolidase with TGF-β substantially improved the diagnostic performance, resulting in an AUC of 0.969. The multivariable biomarker panel also maintained high diagnostic accuracy following internal validation using leave-one-out cross-validation (AUC = 0.892), supporting the robustness of the proposed model. These findings suggest that prolidase may serve as a useful biomarker for the diagnosis of silica-induced pulmonary fibrosis and that its diagnostic performance can be further enhanced when interpreted together with other profibrotic biomarkers. Nevertheless, given the relatively small sample size and experimental design, further validation in larger animal studies and human cohorts is warranted before clinical application.

Beyond its potential as a circulating biomarker, prolidase may also reflect ongoing extracellular matrix remodeling during silica-induced pulmonary fibrosis. As the key enzyme responsible for the final step of collagen degradation, prolidase recycles proline for subsequent collagen synthesis and therefore contributes to collagen turnover and tissue remodeling. Persistent activation of profibrotic mediators, particularly TGF-β, promotes fibroblast activation and excessive extracellular matrix deposition, while PDGF and FGF signaling further amplify fibroblast proliferation and fibrotic progression [10]. Since nintedanib inhibits multiple profibrotic tyrosine kinase pathways, including PDGF and FGF receptors, the observed changes in prolidase activity may indirectly reflect attenuation of fibrotic remodeling rather than collagen degradation alone [14]. Nevertheless, further mechanistic studies are warranted to clarify whether prolidase acts solely as a biomarker or also contributes directly to the pathogenesis of silica-induced pulmonary fibrosis.

This study has several limitations. First, the experimental design was based on a relatively small animal cohort, particularly in the control group, which may limit the statistical power and generalizability of the findings. Second, silicosis was induced using a single-dose intratracheal crystalline silica instillation model, which does not fully reproduce the chronic, repeated, and occupational inhalational exposure pattern observed in human silicosis. Third, although nintedanib significantly improved histopathological injury and collagen deposition, serum prolidase levels did not differ significantly between the untreated silicosis and nintedanib-treated groups; therefore, the role of prolidase as a treatment-response biomarker should be interpreted cautiously. Fourth, biomarker measurements were performed at a single terminal time point, preventing evaluation of dynamic temporal changes in prolidase, TGF-β, FGF-2, and PDGF during disease progression and treatment. Fifth, molecular analyses such as gene or protein expression of profibrotic pathways, oxidative stress markers, and collagen-related signaling were not performed, which limits mechanistic interpretation. Finally, the findings are derived from an experimental rat model and require validation in larger animal studies and human silicosis cohorts before clinical translation.

## 5. Conclusions

In conclusion, this experimental study demonstrated that serum prolidase levels were significantly increased in silica-induced silicosis and showed a strong positive correlation with TGF-β, supporting its association with fibrotic remodeling and extracellular matrix turnover. ROC analysis demonstrated that prolidase has good diagnostic performance for discriminating experimental silicosis from healthy controls, while its combination with TGF-β further improved diagnostic accuracy. Although nintedanib attenuated histopathological lung injury, prolidase levels remained elevated, suggesting that prolidase may reflect ongoing fibrotic remodeling rather than short-term treatment response. These findings support the potential value of prolidase as a promising circulating biomarker for silica-induced pulmonary fibrosis. Further longitudinal experimental studies and clinical validation in patients with silicosis are warranted to confirm its diagnostic and prognostic utility.

## Figures and Tables

**Figure 1:**
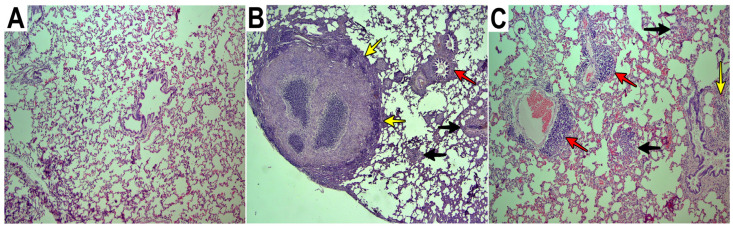
Representative hematoxylin and eosin (H&E)-stained lung sections from the experimental groups (Objective: 10×). (**A**) Control group showing preserved pulmonary architecture with thin alveolar septa, normal alveolar spaces, and no significant inflammatory or fibrotic changes. (**B**) Silicosis group showing a well-demarcated silicotic granulomatous nodule with peripheral fibrosis (yellow arrows), peribronchiolar mononuclear inflammatory cell infiltration (red arrow), and focal interstitial inflammatory infiltrates with alveolar septal thickening (black arrows). (**C**) Silicosis plus nintedanib group showing marked attenuation of pulmonary lesions with largely preserved alveolar architecture, accompanied by mild residual perivascular (red arrows) and peribronchiolar mononuclear inflammatory cell infiltrates (yellow arrows) and focal interstitial inflammatory infiltrates with minimal alveolar septal thickening (black arrows).

**Figure 2:**
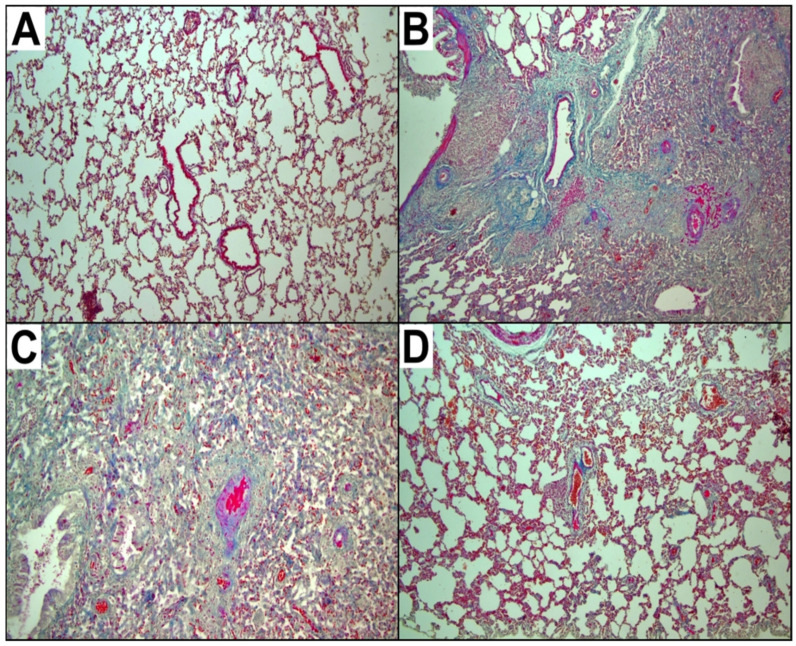
Representative Masson’s trichrome -stained lung sections from the experimental groups (Objective: 10×). (**A**) Control group showing physiological collagen deposition confined to alveolar septa, bronchioles, and vascular walls. (**B**,**C**) Silicosis group demonstrating marked collagen deposition within silicotic nodules and extensive interstitial, peribronchiolar, and perivascular fibrosis. (**D**) Silicosis plus nintedanib group showing markedly reduced collagen deposition with preservation of the pulmonary architecture and only mild residual peribronchiolar and interstitial fibrosis.

**Figure 3:**
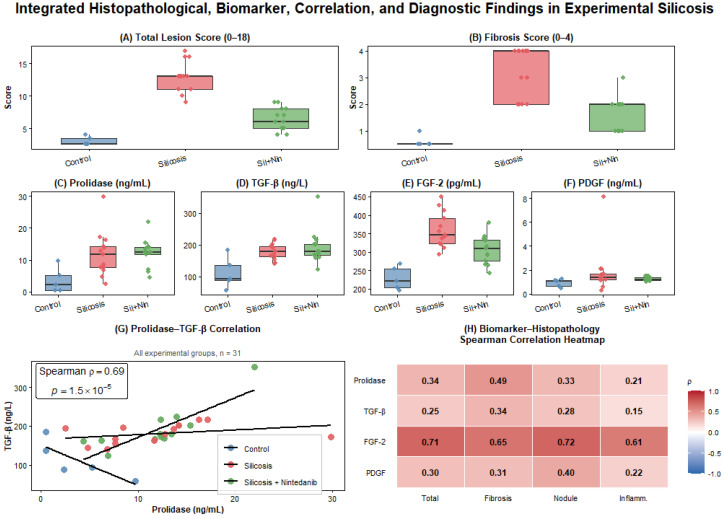
Integrated histopathological, biomarker, correlation, and diagnostic findings in the experimental silicosis model. (**A**) Total lesion score. (**B**) Fibrosis score. (**C**) Serum prolidase levels. (**D**) Serum TGF-β levels. (**E**) Serum FGF-2 levels. (**F**) Serum PDGF levels. (**G**) Correlation between serum prolidase and TGF-β levels in all animals. (**H**) Spearman correlation heatmap showing the relationships between serum biomarkers and histopathological parameters.

**Figure 4:**
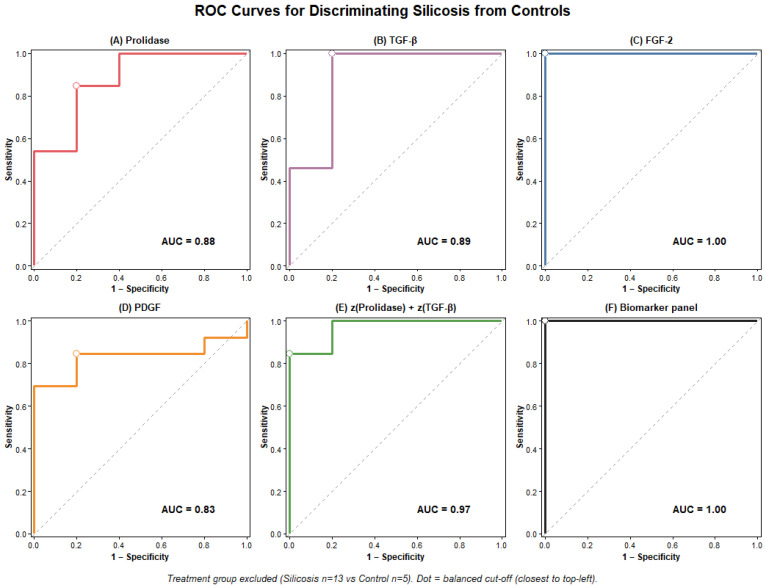
Receiver operating characteristic (ROC) curves for discrimination of silicosis from controls. (**A**) ROC curve for serum prolidase. (**B**) ROC curve for TGF-β. (**C**) ROC curve for FGF-2. (**D**) ROC curve for PDGF. (**E**) ROC curve for the bivariate model based on z(Prolidase) + z(TGF-β). (**F**) ROC curve for the multivariable biomarker panel.

**Table 1:** Comparison of Histopathological Scores Among the Experimental Groups.

Parameter	Control (*n* = 5)	Silicosis (*n* = 13)	Silicosis Plus Nintedanib (*n* = 13)	*p*-Value
Inflammation	1.00 ± 0.00 ^a^	3.15 ± 0.19 ^b^	1.77 ± 0.20 ^a^	<0.001
Mast cell/Eosinophil Density	0.40 ± 0.24 ^a^	1.15 ± 0.15 ^b^	0.23 ± 0.12 ^a^	<0.001
Silicotic Granulomatous Nodule Formation	0.00 ± 0.00 ^a^	3.15 ± 0.15 ^c^	1.15 ± 0.10 ^b^	<0.001
Alveolar Arch. Distortion	1.00 ± 0.00 ^a^	2.31 ± 0.13 ^b^	1.46 ± 0.14 ^a^	<0.001
Fibrosis	1.50 ± 0.88 ^a^	3.23 ± 0.26 ^b^	1.77 ± 0.17 ^a^	0.001
Total Lesion Score	3.00 ± 0.32 ^a^	12.92 ± 0.65 ^c^	6.38 ± 0.49 ^b^	<0.001

Data are presented as mean ± standard error. Different superscript letters within the same row indicate statistically significant differences between groups. One-way ANOVA followed by Tukey’s post hoc test was used for between-group comparisons. A *p*-value of <0.05 was considered statistically significant. Abbreviation: Arch., Architectural.

**Table 2:** Serum Biomarker Levels Among the Experimental Groups.

Variable	Control (*n* = 5)	Silicosis (*n* = 13)	Silicosis Plus Nintedanib (*n* = 13)	*p*-Value
FGF-2 (pg/mL)	221.44 (204.38–253.96) ^c^	345.43 (322.74–392.16) ^a^	309.62 (275.28–332.05) ^a^	<0.001
PDGF (ng/mL)	1.08 (0.68–1.13) ^c^	1.41 (1.20–1.67) ^a^	1.22 (1.16–1.39) ^a^	0.038
Prolidase (ng/mL)	2.34 (0.50–5.27) ^b^	11.64 (7.61–14.17) ^a^	12.36 (11.75–13.97) ^a^	0.017
TGF-β (ng/L)	93.94 (88.47–137.16) ^b^	179.84 (163.04–195.13) ^a^	178.38 (166.82–202.43) ^a^	0.030

Data are presented as median (25th–75th percentile). Between-group comparisons were performed using the Kruskal–Wallis test. Different superscript letters within the same row indicate statistically significant differences between groups. A *p*-value of <0.05 was considered statistically significant. Abbreviations: FGF-2, Fibroblast Growth Factor-2; PDGF, Platelet-Derived Growth Factor; TGF-β, Transforming Growth Factor-Beta.

**Table 3:** Correlation Analysis Between Serum Prolidase and Fibrosis-Associated Proteins.

Variable	Spearman’s Rho	*p*-Value
FGF-2 (pg/mL)	0.20	0.286
PDGF (ng/mL)	0.20	0.271
TGF-β (ng/L)	0.69	<0.001

Spearman’s rho correlation analysis was used to assess the relationships between serum prolidase levels and fibrosis-associated proteins. A *p*-value of <0.05 was considered statistically significant. Abbreviations: FGF-2, Fibroblast Growth Factor-2; PDGF, Platelet-Derived Growth Factor; TGF-β, Transforming Growth Factor-Beta.

**Table 4:** Diagnostic Performance of Prolidase, TGF-β, and Fibrosis-Associated Proteins Combinations for Discriminating Silicosis from Controls.

Model	AUC	Cut-Off	Sensitivity (%)	Specificity (%)
Prolidase	0.877	6.82 ng/mL	85	80
TGF-β	0.892	140 ng/L	100	80
FGF-2	1.000	294 pg/mL	100	100
PDGF	0.831	1.15 ng/mL	85	80
Prolidase + TGF-β (Combined model)	0.969	—	85	100
Apparent Model	1.000	—	100	100
LOOCV Model	0.892	—	92	100

ROC analysis was performed between the control and silicosis groups. Prolidase, TGF-β, FGF-2, and PDGF were evaluated individually, together with the Prolidase + TGF-β combined model and the internally validated multivariable biomarker panel (LOOCV). Abbreviations: ROC, Receiver Operating Characteristic; AUC, Area Under the Receiver Operating Characteristic Curve; FGF-2, Fibroblast Growth Factor-2; PDGF, Platelet-Derived Growth Factor; TGF-β, Transforming Growth Factor-Beta.

## Data Availability

The data supporting the findings of this study are available from the corresponding author upon reasonable request. Due to ethical considerations related to animal experiments, the datasets are not publicly available but can be shared with qualified researchers upon request and approval.

## Abbreviations

AUC	Area Under the Curve
ANOVA	Analysis of Variance
CTGF	Connective Tissue Growth Factor
ELISA	Enzyme-Linked Immunosorbent Assay
FGF-2	Fibroblast Growth Factor-2
H&E	Hematoxylin and Eosin
HSD	Honestly Significant Difference
KL-6	Krebs von den Lungen-6
PBS	Phosphate-Buffered Saline
PDGF	Platelet-Derived Growth Factor
PI3K	Phosphoinositide 3-Kinase
AKT	Protein Kinase B
ROC	Receiver Operating Characteristic
RT-PCR	Reverse Transcription Polymerase Chain Reaction
SPSS	Statistical Package for the Social Sciences
TGF-β	Transforming Growth Factor-Beta
TLR4	Toll-Like Receptor 4
NF-κB	Nuclear Factor Kappa B
VEGF	Vascular Endothelial Growth Factor
